# Microbial metabolism marvels: a comprehensive review of microbial drug transformation capabilities

**DOI:** 10.1080/19490976.2024.2387400

**Published:** 2024-08-16

**Authors:** Filippo Martinelli, Ines Thiele

**Affiliations:** a School of Medicine, University of Galway, Galway, Ireland; b Digital Metabolic Twin Centre, University of Galway, Galway, Ireland; c The Ryan Institute, University of Galway, Galway, Ireland; d School of Microbiology, University of Galway, Galway, Ireland; e APC Microbiome Ireland, Cork, Ireland

**Keywords:** Gut microbiome, microbiota, microbial drug metabolism

## Abstract

This comprehensive review elucidates the pivotal role of microbes in drug metabolism, synthesizing insights from an exhaustive analysis of over two hundred papers. Employing a structural classification system grounded in drug atom involvement, the review categorizes the microbiome-mediated drug-metabolizing capabilities of over 80 drugs. Additionally, it compiles pharmacodynamic and enzymatic details related to these reactions, striving to include information on encoding genes and specific involved microorganisms. Bridging biochemistry, pharmacology, genetics, and microbiology, this review not only serves to consolidate diverse research fields but also highlights the potential impact of microbial drug metabolism on future drug design and *in silico* studies. With a visionary outlook, it also lays the groundwork for personalized medicine interventions, emphasizing the importance of interdisciplinary collaboration for advancing drug development and enhancing therapeutic strategies.

## Introduction

Microbial drug metabolism is a fascinating research field that explores the intricate relationship between microorganisms and pharmaceutical compounds. Microbes, including bacteria, fungi, and other microorganisms, possess remarkable abilities to metabolize various drugs, impacting their effectiveness, toxicity, and pharmacokinetics.^1,2^

Human drug metabolism can be divided into phase I and phase II metabolic reactions. Phase I reactions typically involve the modification of the drug molecule through processes, such as oxidation, reduction, hydrolysis, or dealkylation. These reactions can introduce functional groups or alter the chemical structure of the drug, influencing its pharmacological properties. On the other hand, phase II reactions involve the conjugation of drug metabolites with endogenous molecules, such as glucuronic acid, sulfate, and amino acids. These conjugation reactions increase the water solubility of the drug and facilitate its elimination from the body.^3^

Similarly, microbial drug metabolism involves the biotransformation of drugs through enzymatic reactions, leading to the formation of metabolites that may have altered pharmacological properties compared to the parent drug.^4^ The impact of microbial drug metabolism in medicine cannot be overstated. In some cases, microbial metabolism is essential for the activation of prodrugs, which are inactive or minimally active compounds that require metabolic conversion to exert their therapeutic effects.^5^ Microbial metabolism can also lead to the inactivation or degradation of drugs, reducing their effectiveness, or to the reactivation of specific metabolites elongating the drug effect, or to the production of toxic metabolites.^6^

Microbes have also the ability to indirectly influence drug metabolism by modifying the host’s metabolic processes.^7^ Understanding the direct and indirect interplay between drugs and microbial enzymes is crucial for predicting drug-drug interactions, identifying potential toxicities, designing drugs with enhanced microbial stability, optimizing drug dosing regimens and ensuring their therapeutic efficacy, and developing new therapeutic strategies. The interplay between the host, drug, and microbial factors can lead to significant variability in drug response and treatment outcomes.^8^ Continued research in this field will undoubtedly unlock new avenues for drug discovery and personalized medicine.

Various methods have been employed to explore drug metabolism by the gut microbiota.^6^ *In vitro* culture incubation of the microbes with various drugs is a fundamental approach, in which the microorganism is grown in a controlled environment. This approach allows for the observation of metabolic activities and the identification of specific species involved in the investigated drug metabolic pathway.^9,10^ *Ex vivo* incubation with fecal samples is another valuable technique, especially in studying gut microbiota. By introducing the fecal sample into a suitable culture medium, researchers can analyze the microbial metabolic product and infer their role in specific pathways.^11^ Additionally, *in vivo* studies involving the administration of antibiotics or the comparison of the metabolism of germ-free and control animals allow for the investigation of microbial involvement in metabolic processes.^12^ Furthermore, *in vivo* studies utilizing different routes of drug administration^13^ provide data, on how the microbial community can affect drug metabolism as different administration routes may result in distinct interactions with the microbiota, impacting the overall drug metabolism.

However, these methods have several limitations. *In vitro* cultures might not fully mimic the complex environment of the intestine, showing metabolic abilities that do not accurately represent what would happen *in vivo*. For example, *in vivo* differences in drug uptake and the intestinal milieu can affect outcomes. Oppositely, *in vivo* studies may reveal microbial involvement in metabolism but often fail to identify the specific microbial species and enzymes responsible. Moreover, host factors and interindividual variability can influence results. Consequently, these approaches provide valuable insights into microbial metabolic pathways but necessitate cautious interpretation due to their inherent constraints in representing the intricate *in vivo* conditions and accurately pinpointing the responsible microorganisms and enzymes. A combination of *in vitro*, *ex vivo*, and *in vivo* methods is essential for identifying the microbes responsible for specific metabolic pathways, providing crucial knowledge for advancing our understanding of host-microbe interactions and designing targeted therapeutic strategies.^2^

In recent years, advances in sequencing technologies and bioinformatics have revolutionized the study of microbiome.^14^ Metagenomic approaches have allowed researchers to explore the collective metabolic potential of microbial communities, providing valuable clinical insights into the role of the human microbiome on a larger scale.^15^ For instance, extensive comparative genomic analysis of 25 genes known to encode for 15 enzymes involved in drug metabolism has been performed for 5,438 bacterial strains covering the metabolism of 98 drugs.^16^ The identified drug reactions and pathways were included in genome-scale metabolic reconstructions, which additionally cover comprehensively the biochemical transformation in each strain, as encoded by its genome. This work enabled, for the first time, a large-scale *in silico* investigation of gut microbes and microbial communities involved in drug metabolism. This resource of genome-scale metabolic reconstructions, deemed AGORA2, can also be integrated with organ-resolved, sex-specific, whole-body human metabolic reconstructions,^17^ thereby allowing for future analysis on host-microbiota drug co-metabolism. Such *in silico* analyses, complementing existing *in vitro*, *ex vivo*, and *in vivo* methods, could enhance our understanding of host-microbe interactions, guiding the development of microbiome-modulating therapies to optimize drug outcomes and minimize adverse effects in diverse patient populations. Computational tools and predictive models have been developed to simulate and predict microbial drug metabolism,^18^ possibly aiding in the design and optimization of drug candidates.

Our review, with its extensive collection of gene information based on the extensive comparative genome (re-)annotation work done for the AGORA2 resource,^16^ aims to provide a substantial resource for genetic analysis, such as the BLAST (Basic Local Alignment Search Tool) and GSEA (Gene Set Enrichment Analysis) analysis conducted by Zimmermann,^10^ facilitating the refinement of taxonomic and functional analyses on microbial drug metabolism. Additionally, our comprehensive examination of the drug-transforming reactions and known associated microbes provide a valuable tool for researchers aiming to enhance genomic-scale metabolic models, ensuring a more precise depiction of microbial drug metabolism, and empowering future *in silico* studies. Furthermore, this review seeks to establish a knowledge base for forthcoming drug development studies and interventions in personalized medicine, with a specific consideration of the individual microbiome. This review not only elucidates the current state of knowledge in microbial drug metabolism but also attempts to provide future research with the indispensable tools and insights necessary to advance understanding in the intricate field of drug microbial metabolism.

## Direct effect of microbial metabolism

Direct microbial drug metabolism encompasses a diverse array of reactions, which can be categorized in different ways. From a chemical standpoint, reactions can be classified as azoreduction, deamination, phosphorolysis, hydrolysis, reduction, oxidation, decarboxylation, dehydroxylation, and acetylation. Alternatively, from a pharmacodynamic perspective, the reactions can be classified as activation, modulation, deactivation, toxification, and reactivation, based on the difference in the intrinsic pharmacological activity of the reaction’s reagents and products. Definitions of the chemical and pharmacodynamics terms can be found in Table S1.

Regarding the chemical approach, microbial drug metabolism predominantly involves reduction and hydrolysis reactions, although exceptions exist.^19^ Reductions can have different electron donors and act on various drug moieties, and in some cases, the precise mechanism may not be fully understood due to the absence of genetic and enzyme mechanistic studies.^20^ On the pharmacodynamic side, accurately classifying the reactions is challenging without knowing the specific activity of each produced metabolite.

For this review, we have chosen to organize the reactions based on a univocal classification based on the atoms involved in the broken or formed bond. When possible, additional details regarding the specific chemical aspect and pharmacological activity of each reaction are provided. This classification aims at facilitating the identification of other drugs, whose microbial metabolism remains unexplored, but may undergo similar metabolic pathways, and aid in recognizing the drug moiety that is subject to microbial metabolism. Such insights could prove valuable in evaluating microbial drug metabolism in the development of future pharmaceuticals. A summary of all the reactions described in this section, with associated taxonomical and genetic information, can be found in Table S2.

### C-C bonds

#### Dihydropyrimidine reduction

**Figure 1.**
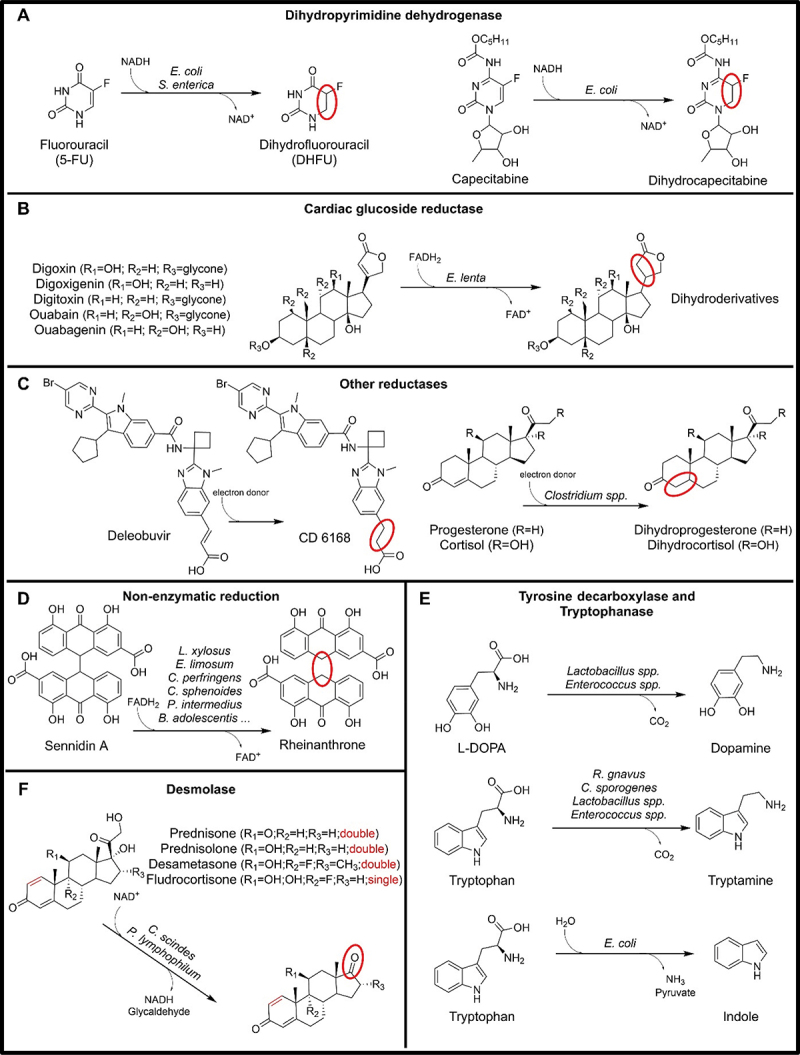
Microbial drug metabolizing reactions involving a C-C bond.

Sixty years ago, bacterial enzymes capable of reductively breaking down pyrimidine have been discovered in *Clostridium uracilicum*.^21^ The first reaction in this catabolic pathway is catalyzed by a dihydropyrimidine dehydrogenase (DPD). Subsequently, researchers have demonstrated the presence of DPD activity in various bacterial strains, such as *Alcaligenes eutrophus*^22^ and *Escherichia coli B*.^23^ Two *Escherichia coli* iron-sulfur proteins, PreT and PreA, have been found to have approximately 30% identity to the *N*- and C-terminal portions of mammalian DPD, respectively, and the *preT* and *preA* genes form an operon structure in the genomes.^24^ The incubation of 5-fluorouracil (5-FU), a chemotherapy drug, with *E. coli* MG1655 and *Salmonella enterica* LT2 showed production of dihydrofluorouracil (DHFU) confirming that the microbial NADH-dependent DPD can also target substituted dihydropyrimidines, such as 5-FU. Additionally, measurements of tumor growth after capecitabine (CAP), a 5-FU prodrug, administration in gnotobiotic mice showed reduced efficacy of the drug in *preTA*^*++*^ *E. coli* colonized mice compared to *ΔpreTA* colonized ones^25^ (Figure 1(A)). In contrast, the activity and specificity of NADPH-dependent DPD of *Pseudomonas aeruginosa* correlated to the *pydX-pydA* genes,^24^ has yet to be tested with the 5FU drug and related compound, such as 5FU prodrugs or compounds with similar structures. The comparative genomic analysis revealed that the DPD enzyme may be present in 935/5,451 distinct strains, spanning 85 species, 35 genera, and three phyla (Table S3).^16^

In conclusion, the NADH-dependent *E. coli* DPD, which is encoded by the *preA-preT* genes, plays a role in 5-FU and capecitabine therapies, by deactivating the metabolite and reducing its cytotoxic effect. The potential implications of *pydX-pydA* NADH-dependent bacterial reductases in this context remain understudied. It is also possible that NADPH-dependent DPDs could also influence the therapies involving other 5-FU precursors, such as tegafur, doxifluridine, floxuridine, and 5-fluorocytosine.

#### Cardiac glycosides reduction

Forty years ago, gastrointestinal bacteria was determined responsible for the reductive metabolism of digoxin to its inactive form dihydrodigoxin.^26^ *Eggerthella lenta* (previously known as *Eubacterium lentum*) has subsequently been identified as the exclusive species responsible for this reductive metabolism. However, the high fecal concentrations level of *E. lenta* alone were not sufficient for the *in vitro* inactivation of digoxin.^27^ Furthermore, *E. lenta* has also been found responsible for the similar reduction of digitoxigenin and its aglycone derivatives, such as digitoxin.^28^ Successive transcriptional profiling, comparative genomics, and culture-based assays revealed a cytochrome-encoding two-gene operon: the cardiac glycoside reductase (*cgr*). This operon is upregulated by digoxin, inhibited by arginine, absent in non-metabolizing *E. lenta* strains, and predictive of digoxin inactivation by the human gut microbiome.^29,30^ Further investigation revealed an expanded 8-gene-cgr associated gene cluster with *cgr2* being sufficient for digoxin activation. The enzyme requires FADH and anaerobic conditions and has been found to reduce other cardiac glycosides, such as digitoxin, digoxigenin the aglycon of digoxin, ouabain and ouabagenin^31^ (Figure 1(B)). The comparative genomic analysis confirmed *E. lenta* as the exclusive microbe having the cgr operon (Table S3).^16^

#### Other C=C reduction

The microbiota can also reduce deleobuvir, an experimental drug for the treatment of hepatitis C, into its main metabolite CD 6168 (Figure 1(C)).^32^ When the drug was incubated with liver microsomes or cytosol, the CD 6168 was not produced, but incubation with rat and human feces resulted in its formation. Furthermore, pseudo-germ-free (pGF) rat models showed a lower rate of reduction metabolism for deleobuvir compared to control rats.^32^ Although the specific microbe or enzyme responsible has not yet been identified, it is crucial to note that this reduction shares similarities with previously described alkene reductions, particularly the presence of an α-β unsaturated carbonylic compound. This similarity suggests that microbes might metabolize drugs with this common moiety through different enzymatic reactions involving electron donors, such as NADH or FADH. Further investigations on microbial reductions on drugs presenting an α-β unsaturated carboxylic acid moiety are essential to evaluate the drugs’ efficacy and for future drug design purposes. Additionally, microbes from the *Clostridium* genera have been found capable of reducing the 4,5 double bond in progesterone^33^ and cortisol^34^ through the action of stereospecific ketosteroid reductases (Figure 1(C)). So far, the responsible genes have not been identified.

#### Dianthrone reduction

It has been shown that different species, such as *Clostridium sphenoides, Clostridium, butyricum, Bifidobacterium adolescentis, Eubacterium rectale, Eubacterium limosum, Egghertella lenta*, and *Peptostreptococcus intermedius* can activate sennosides and sennidins, converting them to rheinanthrone (Figure 1(D)).^35–37^ This activation occurs through an enzymatic reduction carried by sennidin reductase, first isolated from *P. intermedius*.^38^ This enzyme requires NADH as an electron donor and FAD or FMN as electron carriers. However, genes encoding the identified enzyme have yet to be discovered.

#### Aromatic amino acid decarboxylation and degradation

The primary treatment for Parkinson’s disease, the second most common neurodegenerative disease, is levodopa (L-3,4,dihydroxyphenylalanine or L-DOPA). It has been already noted 50 years ago that the gut microflora can decarboxylate L-DOPA.^39,40^ The gene and structure of the enzyme involved in tyrosine decarboxylation have been identified: the enzyme is coded by the *tyrDC* gene, is a pyridoxal-5-phosphate (PLP) dependent tyrosine decarboxylase (TyrDC) and is found in the genome of various bacteria, such as *Lactobacillus* and *Enterococcus*^41–43^ (Figure 1(E)). TyrDC can also decarboxylate levodopa.^42^ Studies linking fecal microbial tyrosine decarboxylases to levodopa levels, clinical variables, and treatment response in Parkinson’s disease^44–46^ prove that the activity of gut microbiota’s TyrDC may interfere with L-DOPA availability and therapeutic efficacy.

Carbidopa, a decarboxylase inhibitor, is commonly used in combination with L-DOPA to reduce decarboxylation of the drug by human enzymes to increase L-DOPA availability,^47^ but it was ineffective against *Enterococcus faecalis* TyrDC.^42^ Instead, (S)-α-fluoromethyltyrosine (AFMT), a tyrosine mimic, has been found to prevent decarboxylation by *E. faecalis’* TyrDC.^42^

Based on comparative genomics, 475/5,451 microbial genomes, encode for the TyrDC enzyme, spanning 20 species, 7 genera, and one phylum, identifying Firmicutes as the exclusive phylum harboring this drug metabolizing capabilities (Table S3).^16^

Additionally, two enzymes from *Clostridium sporogenes* and *Ruminococcus gnavus*, respectively encoded by *CLOSPO_02083* and *RUMGNA_01526* genes, can decarboxylate a different aromatic amino acid, tryptophan, to form the β-arylamine neurotransmitter tryptamine^48^ (Figure 1(E)). Similarly, to the inhibition of TyrDC, (S)-α-fluoromethyltryptophan can inhibit *R. gnavus* tryptophan decarboxylase.^48^ A different degradation of tryptophan is performed by the bacterial enzyme tryptophanase encoded by the gene *tnaA*. The enzyme was first purified from *E. coli*. It is pyridoxal phosphate (PLP) dependent and catalyzes the degradation of tryptophan into indole, pyruvate, and ammonia via α,β-elimination and β-replacement^49^ (Figure 1(E)). No comparative genomic analysis has been carried out for these genes.

#### Side-chain cleavage

Forty years ago, it was recognized that human feces can remove the side chain of cortisol.^50^ Later, *Clostridium scindens* was identified as one of the microbes capable of performing this reaction, with its activity relying on manganese ion and NAD^+^ or NADH.^51^ More recently, the genes *desA* and *desB* have been identified in *C. scindens* as responsible for encoding the corresponding enzyme.^52^ Furthermore, *Propionimicrobium lymphophilum*, a bacterium found in the urinary tract, also carries these genes and has the demonstrated ability to remove the side chain of prednisone, prednisolone, dexamethasone, and fludrocortisone, synthetic pharmaceutical derivatives of cortisol, by releasing glycolaldehyde^34,52^ (Figure 1(F)). Note that the comparative genomics study did not include these gene. This pathway may have implications for glucocorticoid therapy and diseases, such as prostate cancer, as it could potentially contribute as a source of androgens.^53^

### C-N bonds

#### Lactam hydrolysis

**Figure 2.**
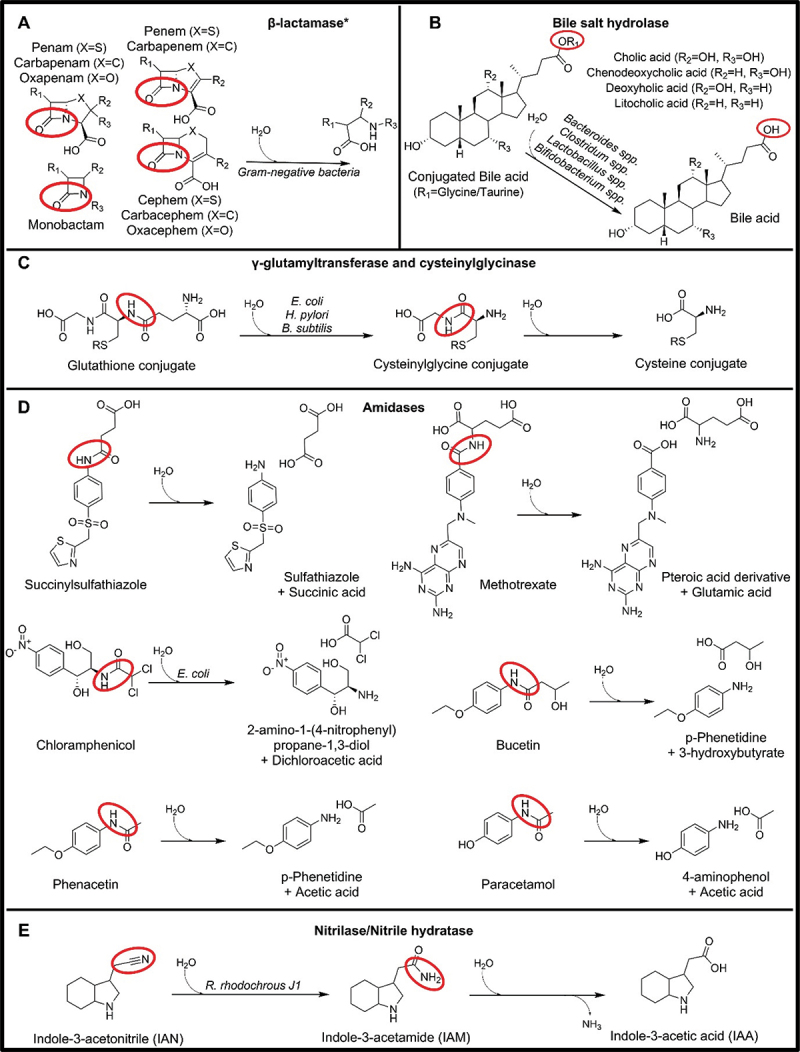
Microbial drug metabolizing reactions involving an amide bond. *β-lactamases activity varies from drug to drug. 2A shows only the core structures of drugs that can be modified by β-lactamases.

Over 80 years ago, it was recognized that a bacterial enzyme was responsible for breaking down penicillin preparations.^54,55^ To-date, we have a deeper understanding that microbial β-lactamases play a significant role in hydrolyzing the amide bond of the four-membered β-lactam ring.^56^ These enzymes are the primary culprits behind the resistance to beta-lactam antibiotics, including penicillins, cephalosporins, carbapenems, and monobactams^56^ (Figure 2(A)). β-lactamases are categorized into four classes: active-site serine β-lactamases (classes A, C, and D) and zinc-dependent or metallo-β-lactamases (MBLs; class B) and different genes (*bla, ctx, tem, ampC, shv, kpc, vim, cmy, adc, oxa*) encode these enzymes, leading to varying substrate specificity.^57^

To combat antimicrobial resistance and broaden the efficacy of antibiotics, a common approach is to co-administer beta-lactam antibiotics with microbial beta-lactamase inhibitors, such as clavulanic acid.^58^ This strategy helps to prevent the degradation of the antibiotics by β-lactamases, thereby extending their spectrum of activity against resistant bacteria.^59^ New microbial MBL inhibitors are constantly investigated and developed to overcome antibiotic resistance.^60^

#### Bile acid amino acid conjugate hydrolysis

Bile acids are natural compounds produced in the liver and stored in the gallbladder. They aid in the digestion and absorption of lipophilic nutrients and drugs in the small intestine.^61^ They play essential roles in cholesterol metabolism and the elimination of waste products from the body.^62^ Bile acid therapy is used for gallstones, but it has also been proven useful in the treatment of primary biliary cholangitis, liver diseases, and liver-associated metabolic diseases.^63,64^

Microbial bile salt hydrolases (BSHs), which catalyze the deconjugation of C-24 amino acid conjugated bile acids, have been isolated and/or characterized from several species of intestinal bacteria^65^ (Figure 2(B)). Homologs and putative *bsh* genes, such as *cbh*, have also been identified.^66^ It has been hypothesized that deconjugation may be a mechanism of the detoxification of bile salts due to their antimicrobial activity.^67^

In a recent study, bile salt hydrolases (BSHs) in the human microbiota have been investigated for their diversity and activity levels across 11 different human populations revealing a widespread presence of BSH protein sequences among intestinal bacteria, with significant variations observed across the populations.^68^ Moreover, Song et al. identified distinct phylotypes of the BSH enzyme in these populations with different activity and distribution, highlighting the diversity and potential functional implications of these microbial enzymes in human health.^68^ Accordingly, the comparative genomic analysis identified the presence of the *bsh* gene in 530/5,451 microbial genomes, spanning 178 species, 62 genera, and six phyla (Table S3).^16,69^

#### Glutathione conjugates amide bond hydrolysis

Glutathione (GSH) conjugation is a common process for many drugs. Both human and microbial enzymes can degrade glutathione conjugate compounds and also glutathione.^70,71^ Studies on gnotobiotic rats and mice have shown the involvement of the microbiota in this process.^72,73^ In this catabolic pathway, the initial two reactions lead to the release of glutamic acid and glycine from the GSH residue. The first reaction is facilitated by the γ-glutamyltransferase enzyme (GGT), encoded by the *ggt* gene, with homologues found in *E. coli, Helicobacter pylori*, and *Bacillus subtilis*.^73–76^ The second reaction involves hydrolysis catalyzed by peptidases with cysteinylglycinase activity.^71^ Peptidase A, B, D, and N of *E. coli*, encoded, respectively, by the *pepA, pepB, pepD*, and *pepN* genes, have shown cysteinylglycinase activity, but no study was conducted on cysteinylglycine drug conjugated compounds.^77^ (Figure 2(C)). No comparative genomic analysis has been carried out for these genes.

#### Other amide bond hydrolysis

Other hydrolysis reactions of amide bonds that are not part of the glutathione degradation pathway can also be performed by gut bacteria. For instance, protein therapeutics, such as azetirelin,^78^ insulin,^79^ and calcitonin,^80^ can be degraded by the intestinal microflora. To avoid microbial degradation, protein drugs are administered intravenously. However, smaller drugs that are administered orally and that have an amide bond can be affected by microbial metabolism. For example, the succinylsulfathiazole prodrug needs to be activated by amidases into sulfathiazole, an antimicrobial for the gastrointestinal tract, with the release of the succinyl group^81^ (Figure 2(D)). Other examples of drugs having an amide bond are methotrexate, which is deactivated with the release of a pteroic acid derivative and glutamic acid;^82,83^ and chloramphenicol, which can be deactivated by *E. coli*^84^ by the gene estDL136^85^ (Figure 2(D)). No comparative genomic analysis has been performed for this gene.

Microbes have also been found to degrade drug molecules that have an amide bond between a carboxylic acid and an aniline as is the case for paracetamol, phenacetin, bucetin, and other acetanilide derivatives^86^ (Figure 2(D)). The breakage of the amide bond occurred in many N-acyl aniline derivatives, although N-benzoyl substituted anilines were not as well deacylated as was the case for anilines para-substituted with aromatic compound or halide groups.^86^ The responsible enzymes involved in this degradation are currently understudied.

#### Nitrile hydration

Nitrile hydratase (NHase) belongs to a class of metalloenzymes that catalyze the transformation of a nitrile compound by acting on its triple bond, converting it first into an amide and then into a carboxylic acid with the release of ammonia (Figure 2(E)). The initial identification of this enzyme occurred in the bacterium *Rhodococcus rhodochrous J1*.^87^ After the discovery, many other microbes, including gut commensals, such as *Enterococcus faecalis* and *Pseudomonas spp*., have been found able to perform similar reactions^88^ and additionally, two different enzymes encoded by the nitrilase (nit) and the nitrile hydratase (nthAB) genes have been found responsible for catalyzing this reaction.^89^ While these enzymes are of great interest for industrial application, their involvement in the metabolism of drugs containing a nitrile group, such as anastrozole, citalopram, and vildagliptin, remains understudied.^90^ No comparative genomic analysis has been carried out for these genes.

#### Cytosine deamination

**Figure 3.**
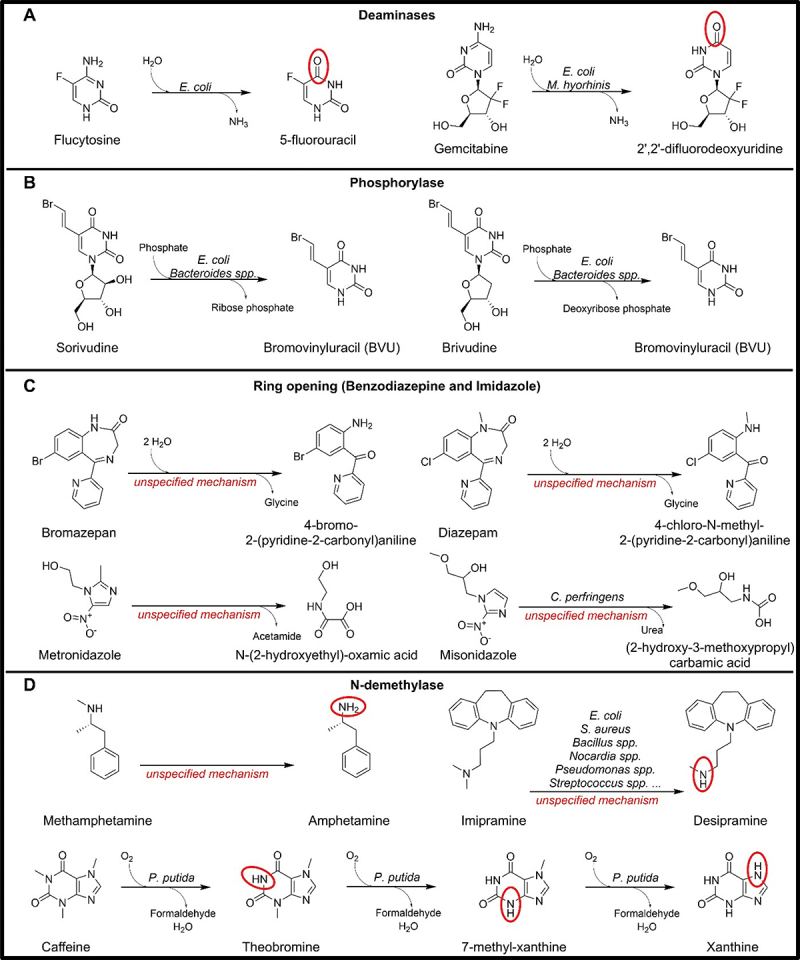
Microbial drug metabolising reactions involving C-N bonds.

Flucytosine, a nucleotide analogue, is an antifungal medication that excerpts its activity after being converted into its cytostatic metabolite 5-fluorouracil after absorption into microorganism cells. The cytosine deaminase enzyme responsible for this amido hydrolyzation was purified from *Saccharomyces cerevisiae*.^91^ Its species-specific metabolism is essential for its antifungal action, but gut microbes can also perform the reaction. The corresponding gene *codA* has been identified in *Escherichia coli*^92^ (Figure 3(A)), and this microbial activity could lead to possible side effects of the drugs.^93^ Novel findings have shown that bacterial deaminases are less efficient in degrading 5-fluoroisocytosine rather than flucytosine, and hence, it is hypothesized that the use of fluoro-isocytosine would produce fewer side effects.^94^ Interestingly, the discovery of cytosine deaminase as a drug-metabolizing enzyme improved cancer therapies. Using purified cytosine deaminase in combination with flucytosine in anticancer treatment instead of 5-fluorouracil by itself led to the reduction of toxic effects, improving the specificity of the toxic metabolite action. Exploring the biochemical properties of the cytosine deaminase and its isoforms could lead to further improvement in cancer therapy.^95^ The comparative genomic analysis identified the cytosine deaminase enzyme across 2,317/5,451 distinct strains, spanning 239 species, 109 genera, and nine phyla (Table S3).^16^

#### Cytidine deamination

Microbial deamination can also happen on nucleoside analogues, such as gemcitabine. *Mycoplasma hyorhinis’* cytidine deaminase is more catalytically active than its human equivalent in the deamination of gemcitabine.^96^ Additionally, deactivation of gemcitabine has been found in incubation with human faces or *Escherichia coli* suspension^97^ (Figure 3(A)). Finally, a study on colon cancer mice showed that Gammaproteobacterial cytidine deaminase expression, encoded by the *cdd* gene, induced gemcitabine resistance showing that intratumor bacteria can contribute to the therapy efficacy.^98^ Based on comparative genomics, 660/5,451 microbial genomes, spanning 660 species, 224 genera, and 12 phyla, carry the *cdd* gene (Table S3).^16^

#### Pyrimidine-nucleosidase phosphorolysis

Nucleoside analogues can also undergo phosphorolysis with the breaking of the bond between the nucleoside and the sugar. This reaction is catalyzed by pyrimidine-nucleosidase phosphorylase, encoded by the *pdp* gene and present in *Escherichia coli* and many *Bacteroides* species, and has been found involved in the metabolism of sorivudine^99^ and brivudine^10^ (Figure 3(B)), with both drugs leading to the formation of the toxic compound bromovinyluracil. While interesting, no study on the bacterial-driven phosphorolysis of other oral-administered nucleoside analogues, such as zidovudine or tegafur, has been performed yet. Overall, the brivudine/sorivudine hydrolyzing enzyme has been found in 189/5,451 distinct strains, spanning 127 species, 18 genera, all belonging exclusively to the Bacteroidetes phylum (Table S3).^16^

#### Ring-opening (benzodiazepine and imidazole)

Incubation with human feces has suggested the ability of the microbiome to hydrolyze the benzodiazepine ring of diazepam and bromazepam, two drugs used in the treatment of anxiety^100^ (Figure 3(C)). Additionally, degradation metabolites of the imidazole ring of metronidazole and misonidazole have been found in the feces of conventional rats while being not detectable in the excretion of germ-free rats.^101,102^ Through an unspecified mechanism, metronidazole is degraded into *N*-(2-hydroxyethyl)-oxamic acid with the probable release of acetamide.^102^ Misonidazole instead is degraded into acetic acid derivatives and urea after the initial reduction of the nitro group to obtain the aminoimidazole (AIM)^101^ (Figure 3(C)). None of the responsible genes or microbes have been identified so far.

#### N-demethylation

N-demethylation by bacteria has been observed for methamphetamine and imipramine showing that N-demethylase activity is common among bacteria^103,104^ (Figure 3(D)). Additionally, demethylation of different purine alkaloids, including caffeine has been observed in *Pseudomonas putida*, and it is attributed to the activity of methylxanthine N-demethylases encoded by *ndm(A/B/C/D)* genes^105^ (Figure 3(D)). No comparative genomic analysis has been performed for this gene. While this transformation has little consequences when the drugs are rapidly absorbed, it could still be of interest for less rapidly absorbed drugs.

### C-O bonds

#### Glycosides hydrolysis

**Figure 4.**
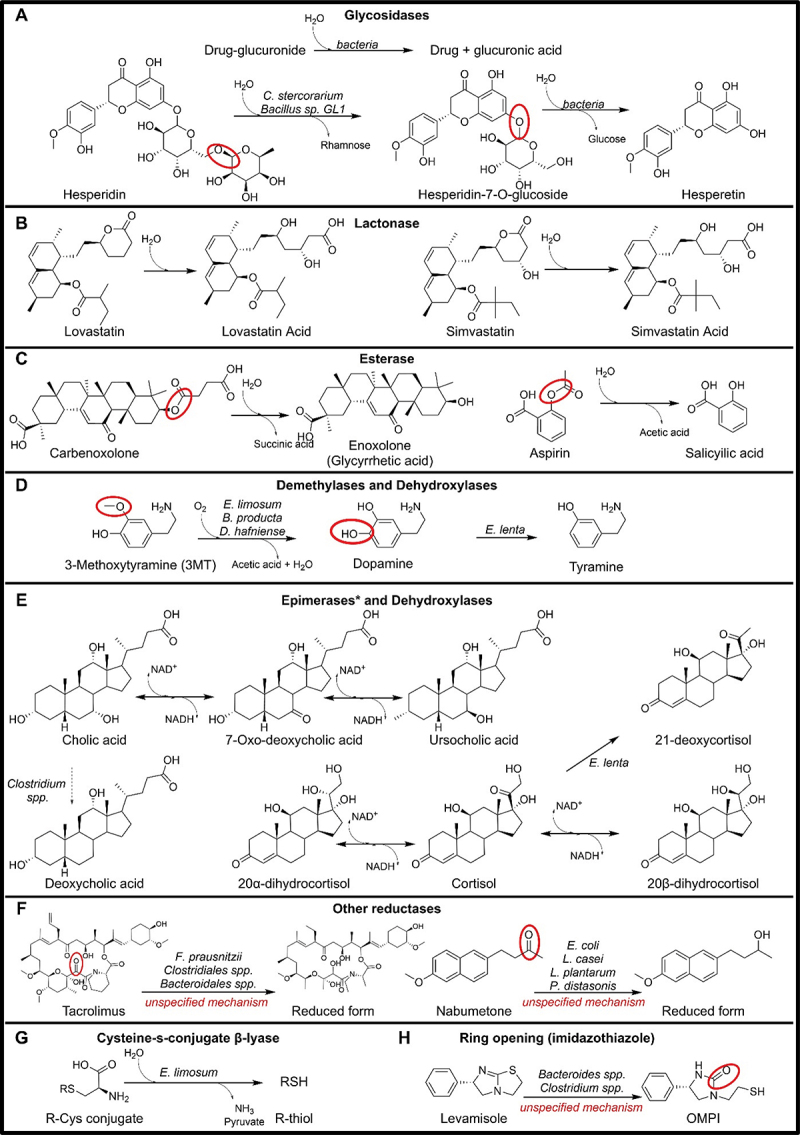
Microbial drug metabolizing reactions involving C-O and C-S bonds. *epimerase activity shown in the picture represents the 7-epimerases acting on cholic acid and the 20-epimerase acting on cortisol. Other epimerases are known to act on hydroxy residues attached to carbon 3,12,17, and 20 of different steroid compounds.

Gut bacteria are known to metabolize non-digestible dietary carbohydrates^106^ and these capabilities can imply the metabolism of glycosidic drugs. Many drugs undergo glucuronidation by human UDP-glucuronosyltransferases to be deactivated and excreted.^107^ Microbes, through the action of β-glucuronidase enzymes, can remove the glucuronide moiety reactivating the drugs and altering their excretion rate (Figure 4(A)). The involvement of the microbe is known in the case of morphine,^108^ camptothecin,^109^ and estrogens.^110^ Moreover, the inhibition of microbial β-glucuronidase by antibiotics or specific inhibitors has been suggested for regorafenib and irinotecan to increase their therapeutic efficacy and reduce their side effects.^111,112^ The microbial gene *uidA*, encoding a β-glucuronidase enzyme, has been first identified in *Escherichia coli*.^113^ Successively, a study on bacterial glucuronidase orthologs identified other genes and microbes that remove the glucuronide moiety from drugs with different substrate efficiency.^114^ Accordingly, comparative genomics analysis identified the uidA gene across 1,553/5,451 microbial genomes, spanning 148 species, 47 genera, and six phyla (Table S3).^16^

Gut microbes can also metabolize lactulose, a prebiotic drug that stimulates the growth of beneficial bifidobacteria and lactobacilli used in the treatment of constipation, degrading it into fructose and glucose. A study involving 453 strains of bacteria commonly found in the human gut identified the gene for lactulose metabolism in 222 of them.^115^ Additionally, the galactosidase could be identified in 2,663/5,451 distinct strains, spanning 505 species, 159 genera, and ten phyla (Table S3).^16^

Other examples of glycosides hydrolysis include the removal of the rhamnosyl moiety of hesperidin by microbial rhamnosidases^116^ as well as other flavonoid rhamnoglucosides, such as narcissin and rutin.^117^ Microbial rhamnosidase structures were isolated from *Clostridium stercorarium*^118^ and *Bacillus sp. GL1*^119^ (Figure 4(A)).

While microbial deglucuronidation could affect the metabolism of all the drugs that are glucuronidated by the host to increase their excretion, the other glycoside hydrolyses are mostly involved in the metabolism of herbal glycosidic drugs, such as sennosides,^36^ rutin, known also as quercetin-glycoside,^120,121^ and calycosin-glycoside,^122^ and their effect on the efficacy of herbal medicines should be considered.

#### Lactone hydrolysis

The microbiome has been found responsible for the lactone ring opening of lovastatin after incubation with human and rat feces while the formation of the active hydroxy acid metabolite decreased in the antibiotic-treated rat^123^ (Figure 4(B)). Other statins, such as mevastatin and simvastatin, are also administered as lactone-ring prodrugs and their activation also requires the opening of the lactone ring and could be influenced by the gut microbiota^124^ (Figure 4(B)). To-date, the responsible genes and microbes have not been identified.

#### Ester bond hydrolysis

The esterase activity of the gut microbiota was already known 50 years ago. By comparing the metabolism of carbenoxolone, a drug used for peptic ulcer disease, in rats between oral and intraperitoneal administration and also by incubating the drug with rat feces, it has been concluded that the gut microflora is responsible for the hydrolysis to glycyrrhetic acid^125^ (Figure 4(C)). However, the responsible genes and microbes remain unknown.

Furthermore, a study on aspirin has shown a prolonged antithrombotic activity in mice when administered with ampicillin, an antibiotic, and human feces incubation of the drug showed the production of its deacetylated derivative, salicylic acid^126^ (Figure 4(C)). While this reaction may be catalyzed by esterases, with aspirin being a phenolic compound, we cannot exclude also the possible involvement of O-acetyltransferases present in many microbes part of the *Staphylococcus, Enterococcus*, and *Lactobacillus* genera.^127^

In the case of diltiazem, a calcium channel blocker, it has been shown that its deacetylation is performed by *Bacteroides thetaiotaomicron* with the *bt4096* gene being responsible for the specific metabolic capability.^10^

#### O-demethylation

O-demethylation by gut microbes has been already evaluated 50 years ago. Through the incubation of different possible substrates with rat cecal microflora, it has been observed that methoxylated benzenoid compounds were demethylated. It has also been noted that compounds resistant to demethylation by the microflora were characterized by the absence of oxy-substitution in positions ortho to the methoxy group.^86^ Successively, many acetogenic bacteria, such as *Eubacterium limosum* and *Blautia producta*, have been identified as responsible for this pathway,^128^ but also non-acetogenic ones, such as *Desulfitobacterium hafniense* DCB-2^129^ and their enzymes were identified as molybdenum-dependent O-methyltransferase.^130^ Interestingly, 3-methoxytyramine has also been identified as a possible substrate of these enzymes, discovering an alternative pathway of acetogenic bacteria to produce dopamine^131^ (Figure 4(D)). Additionally, microbial O-demethylation was also observed for fostamatinib, but only after its initial para-O-demethylation by CYP enzymes suggesting that the presence of the free para-hydroxyl group is a structural requirement for meta-O-demethylation.^132^ Lignans^133^ are also demethylated by gut microbes and we may further hypothesize that this microbial driven O-demethylation may also occur in oral administered etoposide, a chemotherapy drug, due to its lignan-like structure.^134^ None of the responsible genes have been identified so far.

#### Catechol dehydroxylation

The demethylation of a methoxylated benzenoid, which presents an oxy-substitution in ortho to the methoxy group, generates pyrocatechols. These compounds are further metabolized by microbes via catechol dihydroxylation, as is the case for dopamine, a metabolite of levodopa,^135^ (Figure 4(D)), caffeic acid,^73^ fostamatinib,^132^ and lignans.^133^ It has been found that *Eggerthella lenta* molybdenum-dependent dopamine dehydroxylase is responsible for the conversion of dopamine into tyramine^42^ while there are currently no additional insights into the other compounds metabolism. Based on comparative genomics, the dopamine dehydroxylase enzyme could only be found in three distinct strains of two different species: *E. lenta* and *Eggerthella sp. 1_3_56FAA* (Table S3).^16^

#### Other dehydroxylation

*E*. *lenta* also harbors a 21-dehydroxylase,^136^ which can metabolize corticoids, such as cortisol and corticosterone but no responsible enzymes or genes have been identified so far^137^ (Figure 4(E)). Another important dihydroxylation performed by the microbes is the 7a-dehydroxylation of free bile acids. The ability is attributed to the *Clostridium* genera^138^ and *E. lenta*,^139^ which express the bile acid-inducible (*bai*) operon.^140^ In contrast to the straight-forward dopamine dihydroxylation, this specific conversion involves eight different reactions that compose a microbiome-derived pathway that has been recently shown to be able to be expressed and controlled heterologously^140^ (Figure 4(E)). Preliminary comparative genomic analysis on 693 microbial genomes revealed the presence of the bai operon in seven of the analyzed genomes.^69^ The study has also identified new genes belonging to the same cluster and metabolic pathway. *Clostridium scindens’s CLOSCI_00522 and CLOSCI_01264*, respectively ortholog of *E. lenta’s Elen_108* and *Elen_1016* gene, have been suggested to be renamed *baiO* and *baiP*.^69^ In a subsequent comparative genomics analysis, the gene has been identified in 33/5,451 microbial genomes, spanning 18 species, and 14 genera belonging to the Firmicutes and Actinobacteria phyla (Table S3).^16^

#### Hydroxysteroid epimerisation

Microbes can also epimerize hydroxysteroids in a position-specific manner. Two hydroxysteroid dehydrogenases (HSDH) catalyze the oxidation of a specific hydroxy moiety followed by its reduction to generate the correspondent epimer.^65^ Multiple microbes have been identified as producers of 3-, 7-, and 12(α/β)-HSDH responsible for the production of secondary bile acids^141^ (Figure 4(E)). While it has been previously believed that these enzymes could only act on non-conjugated bile acids, a recent study has shown that recombinant HSDHs from a novel identified gene cluster of *E. lenta* were able to utilize taurine and glycine conjugate bile acids as substrates.^142^ The HSDH are position specific and cortisol, a steroid hormone, is a substrate of microbial 3(α/β)-HSDH as well of 17- and 20- (α/β)-HSDH, the latest known also as DesC/E^34^ (Figure 4(E)). Preliminary comparative genomic analysis on 693 microbial genomes revealed the presence of the 7α-, 12α-, 3α-, 3β-, and 7β-HSDHs, respectively, in 46, 39, 17, 12, and three different genomes.^69^ Successive more extensive analysis encompassing 5,451 microbial genomes, confirmed 7α-HSDH to be the most widespread being found in 1,006 distinct strains, spanning 44 species, 18 genera, and six phyla, followed by 12α-HSDH found in 18 species from four different phyla, 3α-HSDH found in 17 species from two different phyla, 3β-HSDH found in 10 species from four different phyla, confirming 7β-HSDH as the rarest, found exclusively in C*ollinsella aerofaciens* and *Mediterraneibacter gnavus* (Table S3).^16^

#### C = O reduction

Microbial drug ketone reduction has been recently observed in the case of tacrolimus, with the reduced metabolite being 15-fold less potent than the immunosuppressant parent drug.^143^ (Figure 4(F)). *Faecalibacterium prausnitzii* was the first bacteria found with this metabolic capability and further screening of other bacteria species has shown that different microbes belonging to the *Clostridiales* or *Bacteroidales* order are also able to metabolize tacrolimus.^143^ Additionally, nabumetone, a nonsteroidal anti-inflammatory drug (NSAID), is reduced and inactivated by gut microbes (Figure 4(F)), in particular by *E. coli, Lactobacillus plantarum, Lactobacillus casei*, and *Parabacteroides distasonis*.^144^ However, the responsible genes remain unknown.

### C-S bonds

#### Cysteine-S-conjugate degradation

Numerous drugs undergo detoxification through conjugation with glutathione (GSH), followed by microbial-mediated hydrolyses, to produce glycine conjugates. These hydrolyses have already been presented in the C-N bond section. Cysteine conjugates can be additionally metabolized into thiol conjugates through the action of cysteine S-conjugate β-lyases that are also present in microbes, such as *Eubacterium limosum*^145^ (Figure 4(G)). This enzyme degrades the cysteine conjugate into a thiol conjugate with the release of pyruvic acid and ammonia, similar to the tryptophanase described in the C-C bond section (Figure 1(E)). The remaining sulfur-containing fragments are normally more reactive than their parent compound, with the reaction representing a bioactivation or toxification mechanism. However, the host can deactivate and eliminate these active compounds through processes, such as methylation or glucuronidation.^146^ The corresponding gene has yet to be identified.

#### Ring-opening (imidazothiazole)

The microbiome can also cleave the C-S bond in the imidazothiazole ring, as in the case of levamisole, resulting in the production of the lactam derivative 2-oxo-3-(2-mercaptoethyl)-5-phenylimidazolidi (OMPI), which may be its active form^147^ (Figure 4(H)). The incubation with individual bacterial strains has shown that the major metabolizing microbes were anaerobic bacteria from the *Bacteroides* and *Clostridium* genera.^147^ The enzyme and mechanism of this reaction are still unclear and while the authors proposed the involvement of an oxidative pathway, due to its activity being seemingly more prevalent in anaerobic bacteria, we would suggest the possible involvement of a substrate-specific hydrolase instead.

### N-N bonds

#### Azo compounds and hydrazones reduction

**Figure 5.**
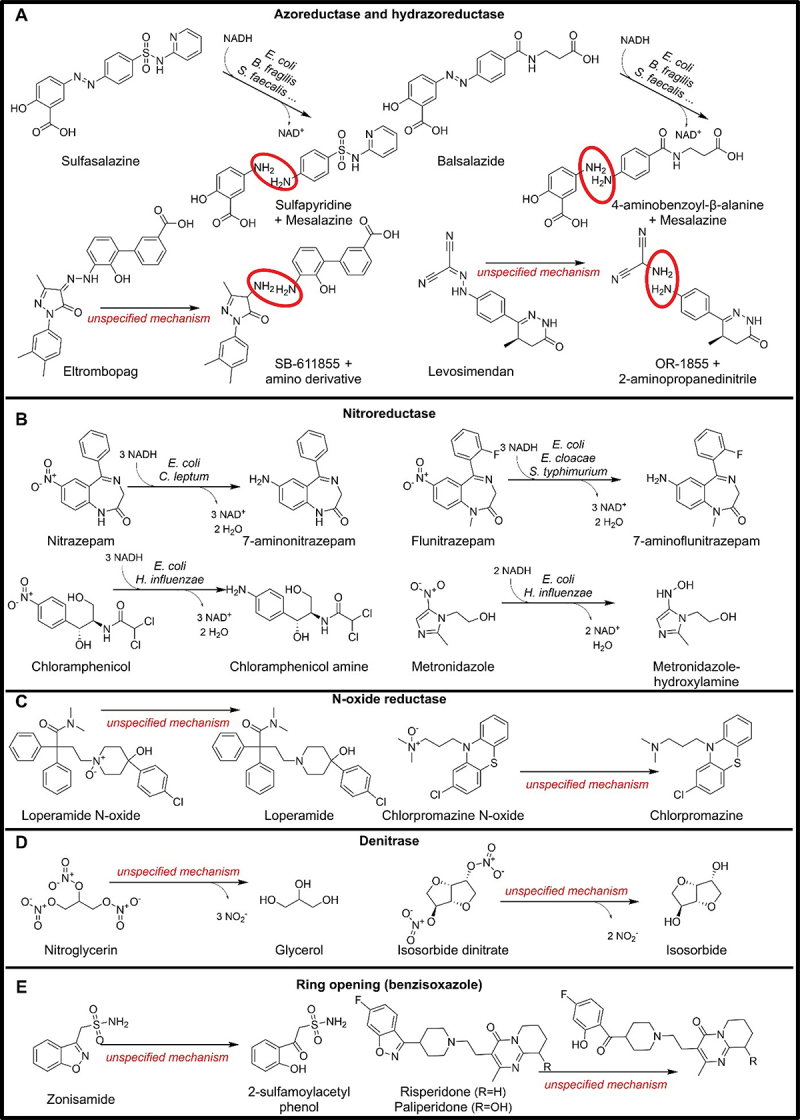
Microbial drug metabolizing reactions involving N-N and N-O bonds.

Gingell et al. have found that rats receiving prontosil or neoprontosil produced less sulfanilamide, a metabolite generated from the azo-bond reduction, when they were treated with antibiotics.^148^ Furthermore, these results were more evident for neoprontosil, as it was more water-soluble and less rapidly absorbed in the gut.^148^ While these two drugs are not commonly used anymore, other drugs containing an azo bond are still on the market. For instance, sulphasalazine is still used to treat autoimmune conditions, such as rheumatoid arthritis and inflammatory bowel disease.^149^ Peppercorn et al. found that while germ-free rats did not metabolize sulphasalazine, conventional rats completely degraded the drug into sulphapyridine and mesalazine, the two products of the azo bond reduction^150^ (Figure 5(A)). Additionally, the finding that gnotobiotic rats infected with four microbial species could degrade the drug further supports the hypothesis that microbes are responsible for the azo-bond reduction.^73^ Subsequently, the crystal structure of the azoreductase (AzoR) isolated from *Escherichia coli* has been obtained, which suggested the requirement of NADH as an electron donor.^151^ Furthermore, its similarity with quinone oxidoreductases suggested that the actual reduction site is not the azo bond itself, but the quinonimine moiety of mesalazine.^152^ While this finding can be extended to other azo-bonded prodrugs, such as balsalazide (Figure 5(A)) and olsalazine,^153,154^ there are still uncertainties about the substrate specificity and the pH sensibility of the enzyme and its isoforms.^155,156^

Additionally, eltrombopag, a drug with a hydrazine bond linked to a quinonimine, can also be metabolized by gut microbes (Figure 5(A)).^157^ We hypothesize that microbial azoreductases are responsible for this degradation. While the azo or hydrazine bond with a linked quinonimine seems important for the substrate specificity of the enzyme, the *E. coli* AzoR can also reduce nitro groups.^158^ Furthermore, the levosimendan hydrazine-bond, even lacking a linked quinonimine moiety, can be reduced by bacteria, with the responsible enzyme being located in the lower parts of the gastrointestinal tract (Figure 5(A)).^159^ The importance of microbial azoreductases does not stop at drug compounds because azo compounds are common in synthetic chemicals, particularly in food dyes and tattoo pigments.^157^

So far, azoreductase has been identified in 78/5,451 microbial genomes spanning 22 species, 15 genera, and three phyla (Table S3).^16^

### N-O bonds

#### Nitroreduction

The high levels of nitroreductase activity in intestinal bacteria are well-documented.^160^ When clonazepam and nitrazepam were exposed to rat intestinal contents under both aerobic and anaerobic conditions, they were rapidly reduced to their amino derivatives.^161,162^ Additionally, pretreatment of the animals with antibiotics decreased nitrazepam-induced teratogenicity, suggesting that the teratogenic effects of nitrazepam are dependent on nitroreduction by the intestinal microflora.^161,162^ The reduction of nitrazepam has been first identified in *Clostridium leptum*.^163^ A successive study of purified *Escherichia coli* recombinant nitroreductase (NfsB) revealed that flunitrazepam, nitrazepam, and clonazepam were all subject to nitroreduction mediated by NfsB (Figure 5(B)). Additionally, two other nitroreductases, from *E. cloacae* NR and from *S. typhimurium* cnr, could also perform the nitroreduction of flunitrazepam.^164^

Nitrobenzodiazepines are not the only drugs that undergo nitroreduction. The inactivation of chloramphenicol through the reduction of its nitro group to an amino group by *E. coli* was identified 70 years ago (Figure 5(B)).^165^ Subsequently, chloramphenicol has also been found to be reduced by *Haemophilus influenzae*’s nitroreductase.^166^ This nitroreductase can confer bacterial resistance to chloramphenicol and perform incomplete reduction of metronidazole (Figure 5(B)) and nitrofurantoin antibiotics.^167^

Taken together, microbial nitroreductases reduce the nitro group of different drugs to an amino group proceeding through a hydroxylamine derivative. The hydroxylamine derivatives are responsible for the antibiotic activity in the case of metronidazole and nitrofurantoin, and the teratogenic activity in the case of nitrobenzodiazepines. The amino derivatives are inactive and this complete reduction lowers the efficacy of the therapies.

Additionally, ranitidine and nizatidine, two H2-receptor antagonists containing a nitro group, are metabolized when incubated with human feces: UV and mass spectrometry analysis indicated that metabolism occurs via cleavage of an N-oxide bond within the molecules with the loss of an oxygen atom.^168,169^ We hypothesize that microbial nitroreductases may be involved in this process.

Notably, microbial nitroreductases have applications beyond drug efficacy evaluation. They are used in gene-directed enzyme prodrug therapy, also known as suicide gene therapy.^170^ By expressing the microbial gene exclusively in cancer cells, the location toxicity of the administered nitro pro-drug can be controlled.^171–173^

Overall, nitroreductases could be identified in 4,206/5,451 microbial genomes, spanning 548 species, 187 genera, and 13 phyla (Table S3),^16^ highlighting their broad distribution in human-associated microbes.

#### N-oxide reduction

Microbes can also perform reduction on N-oxide moieties to produce their respective amines. Loperamide oxide is a prodrug which requires gut microbes to undergo reduction to loperamide, an effective antidiarrheic medication (Figure 5(C)).^174^ Similarly, chlorpromazine N-oxide, an inactive metabolite of chlorpromazine, a psycholeptic drug, can be reactivated by microbes present in the intestinal and urinary tracts of rats (Figure 5(C)).^175^

This pathway may also be relevant in phytotherapy because many alkaloids can have or be metabolized into N-oxide compounds, such as the case of indicine^176^ or nicotine^177^ and its derivatives 4-(methylnitrosamino)-1-(3-pyridyl)-1-butanone (NNK) and 4-(methylnitrosamino)-1-(3-pyridyl)-1-butanol (NNAL).^178^ Additionally, sorafenib (SRF) undergoes activation by CYP3A4, resulting in the formation of sorafenib N-oxide (SRF-Nox), which is utilized as a treatment for renal cell carcinoma.^179^ One can hypothesize that the potential bacterial reduction of the N-oxide in the urinary tract may lead to a decrease in its effectiveness. No specific enzyme has been identified for the reduction of the aforementioned drugs, but we hypothesize an involvement of N-oxide reductases from the same family as the trimethylamine-N-oxide reductase and dimethyl sulfoxide reductase, encoded by *torA* and *dmsA*, respectively, in *Escherichia coli*.^180,181^ No comparative genomic analysis has been carried out for these two genes.

#### Nitrates degradation

Organic nitrates, such as nitroglycerin (glyceryl trinitrate) and isosorbide dinitrate, are used to improve angina pectoris symptoms and can be metabolized into their complete and incomplete alcoholic derivatives by mixed cultures of rat cecal contents (Figure 5(D)).^182,183^ While no specific mechanism has been identified so far, some studies observed that the nitro group was liberated as nitrite, not nitrate, indicating a possible reductive mechanism.^184,185^ The liberated nitrite is recycled in blood and tissue to form nitric oxide (NO) that retains the pharmacological action of the drugs,^186^ so the microbial involvement could be significant for the activation of these drugs and their efficacy. The responsible microbial genes remain unknown.

#### Ring-opening (benzisoxazole)

Zonisamide is metabolized through benzisoxazole ring reduction to form 2-sulphamoylacetylphenol.^187^ A study involving antibiotic-treated rats suggested the involvement of intestinal bacteria in this reductive conversion. Using cell-free extracts of *Clostridium sporogenes*, significant drug-metabolizing activity has been observed and NADH or NADPH-dependent reduction of zonisamide has been demonstrated (Figure 5(E)).^187^ Risperidone and paliperidone, two antipsychotics with a benzisoxazole moiety, also undergo benzisoxazole ring scission in their metabolic pathway in humans.^188^ Bacterial involvement in their metabolism has been proven through a stability experiment, in which the drugs were incubated with sterile and bacterially inoculated porcine blood. While the drugs were stable in the sterile culture, they were degraded into their 2-hydroxybenzoyl derivatives in the bacterial culture (Figure 5(E)).^189^ The responsible genes are currently not known.

### S-O bonds

#### S-oxide reduction

**Figure 6.**
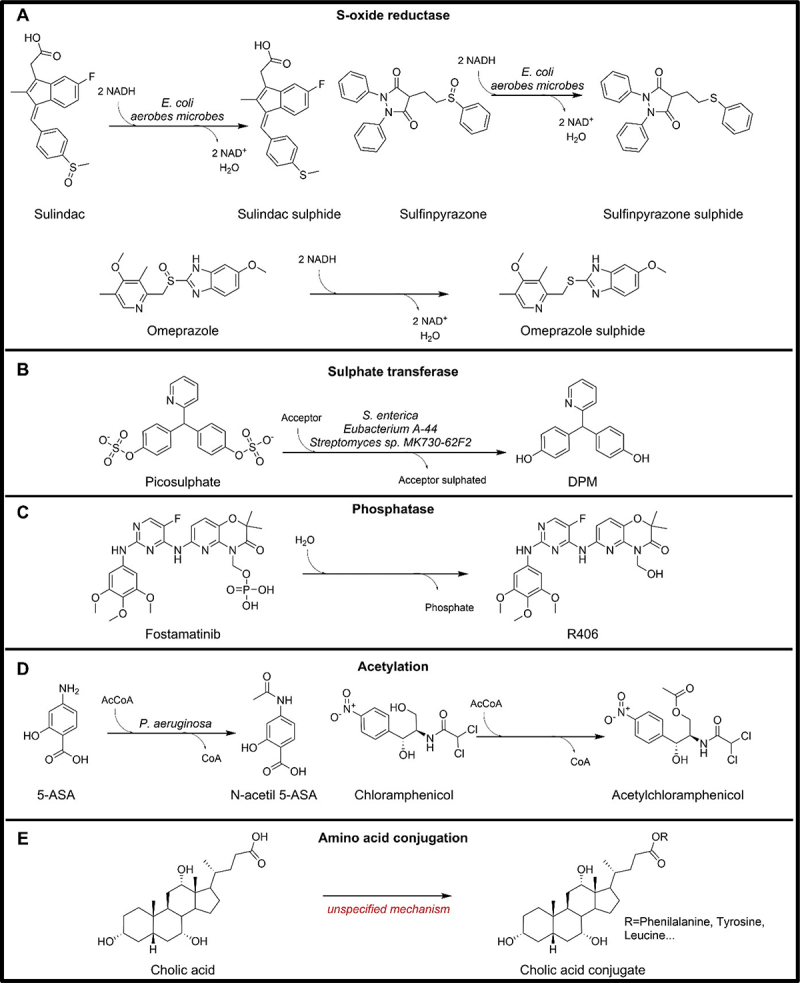
Microbial drug metabolizing reactions involving S-O, P-O bonds, and conjugations.

Forty years ago, through a study of the metabolism of sulfinpyrazone in germ-free and antibiotic-treated rats, the involvement of the gut microbiota in the reduction of the S-oxide moiety of the drug was suggested.^190^ Bacterial culture proved that *Arthrobacter sp*. ATCC 19,140 was able to reduce sulindac S-oxide moiety to sulfide (Figure 6(A)).^191^ Further confirmation of the reductive metabolism was obtained through the incubation of sulfinpyrazone with human feces. It has been observed that the formation of sulindac and sulfinpyrazone sulfides was reduced in patients treated with metronidazole, an antibiotic and antiprotozoal medication. Additionally, screening of over 200 strains of bacteria isolated from human faces showed different reductive potential for the two drugs with both being reduced by mostly aerobic microbes (Figure 6(A)).^192^ A study on *Escherichia coli* reductive potential showed the presence of different sulfoxide reductase systems with different substrate specificity and different cofactor requirements.^193^ By testing different members of the thioredoxin-dependent methionine sulfoxide reductase (Msr) family, MsrA has been found to reduce sulindac to its active sulfide,^194^ but other enzymes that are part of the molybdenum family can also reduce S-oxides and they have yet to been evaluated regarding their drug S-oxides reduction potential.^195^ No comparative genomics analysis has been carried out for MsrA.

Other drugs, such as omeprazole, as well as other prazoles containing the S-oxide moiety, may also undergo S-oxide reduction by gut microbiota (Figure 6(A)).^196^ The importance of this metabolic pathway is not only limited to the activation of S-oxide drugs but encompasses all the drugs that contain a sulfide group, which can undergo oxidation by CYP enzymes. The restoration of the initial sulfide group through reduction by gut microbiota has the potential to extend the activity of the drugs.

#### Sulphate transfer

Sodium picosulphate undergoes conversion into 4,4’-dihydroxydiphenyl-(2-pyridyl)methane (DPM), a crucial component of the laxative (Figure 6(B)). Incubation with rat faces revealed the necessity of phenol for this transformation, indicating the involvement of a sulphotransferase enzyme that requires phenolic acceptors. In support of this finding, the reaction has been successfully catalyzed by a purified sulphotransferase from *Eubacterium* A-44.^197^ Sulphotransferases have been identified in *Streptomyces sp*. MK730-62F2^198^ and *Salmonella enterica* serovar Typhi IMSS-1,^199^ with the *assT* gene encoding it in the latter. While the sulfate moiety is not common, many drugs undergo sulfation by the host to improve their hydrophilicity and subsequent elimination. The microbial reaction is significant for the activation of picosulphate and can also potentially modify the excretion pattern of drugs undergoing host sulfation, as it affects their hydrophilicity and elimination. No comparative genomics has been performed for the assT gene.

### P-O bonds

#### Phosphate hydrolysis

Fostamatinib is rapidly hydrolyzed into its active metabolite R406 by the alkaline phosphatase in the gut (Figure 6(C)).^132^ Alkaline phosphatases occur widely in nature, including in humans and bacteria.^200^ While no specific microbes have been experimentally shown to activate fostamatinib, the alkaline phosphatase, encoded by microbial gene *phoA*, could be identified in 2,618/5,451 microbial genomes, spanning 325 species, 115 genera, and 12 phyla (Table S3).^16^

### Conjugations

The microbial involvement in phase II reactions of the overall drug metabolism is an important aspect of microbial drug metabolism.^6^ Human phase II reactions typically involve the conjugation of drugs or their metabolites with endogenous compounds, such as glucuronic acid, sulfate, amino acids, or glutathione.^201^ Some bacteria possess sulphotransferases or acetyltransferases that can catalyze the addition of sulfate or acetyl groups to drugs or their metabolites, respectively.^202,203^ The microbial involvement in phase II metabolism affects the clearance rate and bioavailability of drugs. Hence, this microbial drug metabolism can have significant implications for drug effectiveness, toxicity, and overall pharmacokinetics.

#### Acetylation

Acetylation of sulphapyridine and mesalazine (5-aminosalicylic acid, 5-ASA) has been observed in the feces of rats, guinea pigs, dogs, and humans (Figure 6(D)).^204^ The N-acetyltransferases enzyme (NAT) encoded by the *nat* gene has been identified and investigated in different bacterial species. It has been found that bacterial NAT activities are comparable to the human NAT1 with both having higher catalytic efficiency with 5-ASA than its isomer 4-ASA.^205^ NAT from *Pseudomonas aeruginosa* showed additional activity on the anti-tubercular drug, isoniazid, as well as on p-aminobenzoic acid (PABA), a folate precursor.^206^ Recently, a novel acetyltransferase, encoded by the gene *nhoA*, has been identified in *Enterobacter sp*. strain CZ-1.^207^ Bacterial N-acetyltransferases are also responsible for the acetylation of other substituted anilines, such as the eltrombopag reduction metabolite, SB-611855, whose acetylated form has been found after incubations with both rodent cecal contents and human feces.^157^ Moreover, the comparative genomics analysis identified NAT encoding genes in 2,165/5,451 distinct strains, spanning 100 species, 32 genera, and four phyla (Table S3).^16^

Taken together, bacterial NATs influence drug metabolism within the gut microbiota, affecting drug activity. Understanding these interactions may lead to personalized medicine advancements and improved therapeutic strategies for various clinical conditions.

Additionally, microbial enzymes can perform O-acetylation. For example, the chloramphenicol acetyltransferase (CAT) encoded by the *cat* gene in *E. coli* and other Gram-negative bacteria has been shown to catalyze the acetyl-S-CoA-dependent acetyl at the 3-hydroxyl group (Figure 6(D)).^203^ No comparative genomics has been carried out for this gene.

Finally, gene *bt_2367* has recently been shown to have acyltransferase properties converting periciazine to acetyl- and proprionylpericiazine.^10^

#### Bile acids amino acid conjugation

Microbes do not only perform hydrolysis on the glycine and taurine-conjugated bile acids produced by the host, but they are also able to conjugate bile acids with other amino acids. Phenylalanine, tyrosine, and leucine conjugates have been identified in a study comparing metabolomics data from germ-free and specific pathogen-free mice (Figure 6(E)).^208^ More recently, through a synthesis-based reverse metabolomics approach, further amino acid conjugates and bile acids have been identified in human feces additionally showing a difference in their level in Crohn’s disease compared to healthy controls.^209^ However, the responsible genes have yet to be identified. This significant expansion of bile acid conjugates has the potential to revolutionize our view on the role of microbial metabolism in host-related diseases.

#### Sulphation

We already discussed sulphotransferases for their involvement in the removal of the sulfate group from sodium picosulphate (Figure 6(B)). They may also induce the sulfation of drugs that have phenolic moieties. Drugs, such as paracetamol, can act as a substrate acceptor of microbial sulphotransferases.^197^ While sulfation is already performed by the host, it cannot be excluded that the microbes could also perform the same reaction thereby further increasing the excretion rate of the drug and reducing its bioavailability.

## Indirect effect of microbial metabolism

The microbiome also has the potential to indirectly influence drug metabolism by modifying the conditions within the gut. The presence of gut microbes leads to the production of metabolites that can affect the integrity of the intestinal barrier, influence drug transporters, and interact with drug-metabolizing enzymes.^7^ As a result, the way drugs are processed in the body, known as pharmacokinetics, is impacted. Additionally, the microbiome plays a role in altering gut pH^210^ and engaging in competition for nutrients,^211^ which, in turn, can affect the solubility, bioavailability, and stability of drugs.

### Alteration of drug metabolism

The microbiome can indirectly affect drug metabolism by producing metabolites that inhibit drug-metabolizing enzymes. For instance, p-cresol is produced by *Clostridium difficile* through the fermentation of tyrosine.^212^ The metabolite is then sulfated by human sulphurtransferases, thereby acting as a competitive inhibitor for the sulfonation of other metabolites.^213^ Many drugs undergo sulfonation and in the case of paracetamol, p-cresol sulfate levels in urine could be correlated to the paracetamol-sulfate/paracetamol-glucuronide ratio suggesting a microbial-driven metabolic switch in the drug metabolism.^214^ Overall, the 4-hydroxyphenylacetate decarboxylase, the enzyme producing p-cresol, has been found in 32/5,451 distinct strains, spanning seven species, six genera, and three phyla (Table S3).^16^

Similarly, the microbiome can influence drug-drug interaction, as is the case for the production of bromovinyluracil (BVU) from the antiviral drugs sorivudine and brivudine.^99^ BVU is an inhibitor of the human dihydropyridine dehydrogenase acting on 5-fluorouracil (5FU).^215,216^ This interaction was responsible for the death of 18 patients that received sorivudine during chemotherapy with tegafur, a 5FU prodrug^217,218^ as well as the death of a patient who received brivudine while being treated for metastatic colorectal cancer with capecitabine, another 5FU prodrug.^219^ BVU can be produced by both host and microbial metabolism. Their contribution has recently been disentangled showing that microbial activity accounts for nearly all the serum BVU measures at later timepoints (>3 h), and 71% of total BVU exposure in the serum of mice. These results strongly suggest a leading role of the microbiome in deathly outcomes.^10^

### Alteration of bioavailability

The microbiome can also affect drug bioavailability without modifying the drug structure by altering the solubility, absorption, and transport of drug compounds.^220^ As described in the previous sections, the gut microbiome produces secondary bile acids and can hydrolyze conjugated bile acids into their free form.^69,221^ In particular, gut microbiome modulation of drug pharmacokinetics through regulation of P-gp has been demonstrated in the case of tacrolimus.^222^ The microbiome can also affect OATP2B1, a transporter that mediates the absorption of different drugs including statins,^223^ limiting the transporter food-induced inhibition, and rescuing intestinal drug absorption.^224^ Additionally, gut microbiota can produce short-chain fatty acids (SCFA),^225^ which are known to increase transepithelial resistance (TER) in Caco-2 cells^226^ and are a potential supplementation therapy to restore gut permeability in people with liver disease.^227^ This permeability alteration could affect drugs’ passive absorption in the intestinal epithelial cells. Finally, gut microbes can bioaccumulate drugs, such as duloxetine, altering their availability.^9^ All these alterations could participate in pharmacokinetics, impacting drug response, and possibly causing adverse side reactions or therapy failure.

## Therapeutical inhibition of microbial drug-metabolizing capabilities

Beta-lactamase inhibitors are crucial in combating antibiotic resistance by blocking bacterial enzymes that degrade beta-lactam antibiotics. They were introduced into therapy almost 50 years ago with clavulanic acid being the first beta-lactamase inhibitor,^228^ and they restore antibiotic efficacy, broaden their spectrum, and preserve their usefulness against resistant strains, thus, representing a vital tool in the fight against drug-resistant infections.^57^

Beta-glucuronidase inhibitors play a significant role in pharmacology and drug development. These enzymes are responsible for cleaving glucuronic acid conjugates, thereby reactivating drugs or metabolites excreted into the gut. By inhibiting beta-glucuronidases, the recycling of drugs could be minimized, reducing potential toxicity and drug reabsorption.^229,230^ Understanding and utilizing beta-glucuronidase inhibitors offer promising avenues for improving drug safety, efficacy, and precision in therapeutic interventions.^231^ In particular, in the case of irinotecan, microbial beta-glucuronidases have been proposed as a possible biomarker to predict irinotecan-induced diarrhea.^232^ Both probiotics and bacterial beta-glucuronidase inhibitors have been shown to reduce the drug’s gastrointestinal toxicity^111,233,234^

Additionally, carbidopa plays a crucial role in Parkinson’s disease therapy. It is a peripheral decarboxylase inhibitor that prevents the conversion of levodopa to dopamine in the bloodstream, allowing more levodopa to reach the brain. Unfortunately, carbidopa fails to prevent L-dopa decarboxylation by *E. faecalis;* therefore, new specific molecules, such as (S)-α-fluoromethyltyrosine,^42^ are required to inhibit gut microbial L-dopa metabolism.

## Conclusion

Personalized therapies that assess microbial drug-metabolizing capabilities hold immense potential to reduce variability in treatment outcomes. By understanding an individual’s gut microbiota and its enzymatic capabilities, clinicians could tailor drug treatments to address an additional layer of individual variability in drug response (IVDR) to reduce adverse drug reactions (ADR) and optimize drug efficacy.^235^ Recognizing the role of microbial metabolism holds paramount importance in preempting potentially lethal drug-related repercussions, as illuminated by instances such as the co-administration of brivudine and fluorouracil. The microbial transformation of brivudine into bromovinyluracil, a dihydropyrimidine dehydrogenase (DPD) inhibitor, accentuates the toxic impact of fluorouracil, exemplifying life-threatening ramifications.^236^ Additionally, the co-administration of cytochrome inhibitors to increase clinical exposure of drugs that undergo cytochrome degradation could lead to metabolic switching and generation of currently understudied microbial products.^237^ Grasping the intricate interplay between drug metabolism and the gut microbiota is pivotal for customizing treatment regimens, sidestepping unwarranted drug interactions, and mitigating the peril of adverse outcomes or therapy failure.^235^ Infusing microbiome analysis into drug development or clinical protocols holds the potential for upholding patient safety and optimizing treatment efficacy.^238^

An *in silico* analysis across 616 microbiomes, using the AGORA2 resource, has illustrated the interpersonal variation of microbial drug metabolism as a function of the microbial composition.^16^ While most drugs could be converted by the studied microbiomes, quantitative differences have been predicted and could be correlated to BMI, age, and sex. Furthermore, the microbial activity against balsalazide, digoxin, and levodopa was dependent on the presence of *E. lenta* in the microbiomes and thus these drug conversions were only present in a subset of the studied microbes.^16^ Such *in silico* investigation, particularly when human drug metabolism is also simulated using whole-body models of human metabolism,^17^ could be used to identify candidate subsets of patients, harboring specific microbes, for optimal trial design, for identifying *in silico* patients suitable for a particular drug treatment where alternatives exist, as well as predicting consequences of multi-drug treatment and drug-diet interactions.^239^ As such, the enumeration of drug-metabolizing capabilities and their addition to computational models represents a pivotal step toward personalized medicine.

This review highlights the impact of the microbiome on drug metabolism. However, it’s essential to recognize the bidirectional nature of these interactions as drugs can also influence the composition of the microbiota.^240,241^ For instance, chemotherapy, proton pump inhibitors, and immunomodulatory drugs have demonstrated such effects.^242^ These alterations can affect drug efficacy, toxicity, and patient responses. Understanding these bidirectional relationships between drugs and the microbiome is thus crucial for optimizing therapeutic outcomes.^243^

## Supplementary Material

Supplemental Material

## Data Availability

This is a literature review
